# 

**Published:** 1896-03

**Authors:** 


					CONTENTS.
Editorial Note
Induced Premature Labour in cer-
tain Diseases of the Mother not
Obstructing Delivery.
By J. G.
Swayne, M.D.Lond.
The Use of Alcohol from a Medical
Standpoint.
By John Dacre,
M.R.C.S. Eng., L.R.C.P. Lond.
10
Two Years' Experience of the Opera-
tion for Strangulated Hernia at
the Bristol General Hospital:
and its Lessons.
By Charles A.
Morton, F.R.C.S.Eng.
23
Extra - peritoneal Suppuration in
Perforating Appendictis.
By J.
Lacy Firth, M.D., M.S. Lond.,
F.R.C.S. Eng
36
Notes on a Case of Exophthalmic
Goitre.
By F. H. Edgeworth, M.B.,
B.A. Cantab., B.Sc. Lond.
41
Progress of the Medical Sciences:
Medicine.
By Theodore Fisher,
M.D.
47
Surgery.
By W. M. Barclay.
53
Ophthalmology.
By F. R. Cross,
M.B.
67
Reviews of Books
71
Notes on Preparations for the Sick
87
Meetings of Societies
91
The Library of the Bristol Medico-
Chirurgical Society 104
Local Medical Notes
110
Scraps
113

				

